# Supplementary material for: Volume electron microscopy reveals unique laminar synaptic characteristics in the human entorhinal cortex
Source: eLife. 2025 Jan 30;14:e96144. doi: 10.7554/eLife.96144 (PMC11867616; doi:10.7554/eLife.96144)
Supplement: Supplementary file 1. — (a) Light microscopy data: volume fraction occupied by cortical elements in layers I, II-is, II-ni, III, Va/b, Vc, and VI of the MEC. Vc: volume fraction occupied by cells bodies; Vbv: volume fraction occupied by blood vessels; Vn: volume fraction occupied by neuropil. (b) Light microscopy data: volume fraction occupied by cortical elements in layers I, II-is, II-ni, III, Va/b, Vc, and VI of the MEC, for individual cases. Vc: volume fraction occupied by cells bodies; Vbv: volume fraction occupied by blood vessels; Vn: volume fraction occupied by neuropil. (c) Summary of the stack details obtained from the multiple sampling from all layers of MEC. Three stacks of images were acquired in each MEC layer, per case (nine stacks of images in total per layer). The last row shows the sum of all the MEC layers from all cases. (d) Accumulated data acquired from the ultrastructural analysis of neuropil from layers I, II-is, II-ni, III, Va/b, Vc, and VI of the MEC for individual cases. Data in parentheses are not corrected for shrinkage. AS: asymmetric synapses; CF: counting frame; SS: symmetric synapses. (e) Number of synaptic SAS analyzed (n), the location (µ), and scale (σ) of the best-fit log-normal distributions in the six cortical layers. AS: asymmetric synapses; SAS: synaptic apposition surface; SS: symmetric synapses. (f) Proportion of the different shapes of synaptic junctions in MEC layers. Data in parentheses refer to absolute numbers of synapses. (g) Proportion of the different shapes of synaptic junctions in MEC layers for individual cases. Data in parentheses refer to absolute numbers of synapses. (h) SAS area of asymmetric (AS) and symmetric (SS) synapses for each synaptic shape in each MEC layer. Data in parentheses are not corrected for shrinkage. Values (in nm2) are expressed as mean ± SE. (i) SAS area of asymmetric (AS) and symmetric (SS) synapses for each synaptic shape, in each MEC layer, per individual case. Data in parentheses are not corrected for shrink [file elife-96144-supp1.docx]

Supplementary File 1 for

Volume Electron Microscopy Reveals Unique Laminar Synaptic Characteristics in the Human Entorhinal Cortex

Sergio Plaza-Alonso, Nicolás Cano-Astorga, Javier DeFelipe and Lidia Alonso-Nanclares

*Corresponding Author: L. Alonso-Nanclares // E-mail: aidil@cajal.csic.es

**This File includes:**

Supplementary File 1a-p

References for Supplementary File 1o

**Other Supplementary Materials for this manuscript include the following:**

Figure 13 – Video 1

Supplementary File 2

Supplementary File 3

Supplementary File 4

| **Layer** | **V_c_ (%±SD)** | **V_bv_ (%±SD)** | **V_n_ (%±SD)** |
| --- | --- | --- | --- |
| **I** | 1.40±0.43 | 2.70±0.62 | 96.01±0.25 |
| **II-is** | 9.20±0.66 | 2.69±1.45 | 88.11±2.03 |
| **II-ni** | 3.13±1.04 | 4.38±0.43 | 92.49±1.13 |
| **III** | 9.26±0.91 | 5.53±1.74 | 85.71±0.83 |
| **Va/b** | 8.97±1.05 | 3.57±0.45 | 87.46±1.07 |
| **Vc** | 6.80±1.25 | 2.86±0.11 | 90.34±1.33 |
| **VI** | 6.60±0.75 | 3.99±1.27 | 89.41±2.02 |

Supplementary File 1a. Light microscopy data: volume fraction occupied by cortical elements in layers I, II-is, II-ni, III, Va/b, Vc and VI of the MEC. Vc: Volume fraction occupied by cells bodies; Vbv: Volume fraction occupied by blood vessels; Vn: Volume fraction occupied by neuropil.

| **Layer** | **Case** | **V_c_ (%)** | **V_bv_ (%)** | **V_n_ (%)** |
| --- | --- | --- | --- | --- |
| **I** | **AB2** | 1.82 | 2.01 | 96.17 |
|  | **AB7** | 0.97 | 3.20 | 95.83 |
|  | **M16** | 1.42 | 2.88 | 95.70 |
| **II-is** | **AB2** | 9.75 | 4.33 | 85.92 |
|  | **AB7** | 9.40 | 2.11 | 88.49 |
|  | **M16** | 8.46 | 1.62 | 89.92 |
| **II-ni** | **AB2** | 3.68 | 4.82 | 91.50 |
|  | **AB7** | 3.78 | 3.97 | 92.25 |
|  | **M16** | 1.93 | 4.35 | 93.72 |
| **III** | **AB2** | 9.70 | 4.70 | 85.60 |
|  | **AB7** | 8.21 | 7.48 | 84.31 |
|  | **M16** | 9.87 | 4.31 | 85.82 |
| **Va/b** | **AB2** | 7.92 | 3.40 | 88.68 |
|  | **AB7** | 10.03 | 3.24 | 86.73 |
|  | **M16** | 8.95 | 4.08 | 86.97 |
| **Vc** | **AB2** | 8.01 | 2.98 | 89.01 |
|  | **AB7** | 6.87 | 2.79 | 90.36 |
|  | **M16** | 5.51 | 2.83 | 91.66 |
| **VI** | **AB2** | 7.36 | 5.35 | 87.28 |
|  | **AB7** | 5.86 | 2.85 | 91.30 |
|  | **M16** | 6.58 | 3.76 | 89.67 |

Supplementary File 1b. Light microscopy data: volume fraction occupied by cortical elements in layers I, II-is, II-ni, III, Va/b, Vc and VI of the MEC, for individual cases. Vc: Volume fraction occupied by cells bodies; Vbv: Volume fraction occupied by blood vessels; Vn: Volume fraction occupied by neuropil.

| **Layer** | **Case** | **No. of stack of images** | **Total volume of Neuropil (μm^3^)** | **No. of images per stack (range)** | **Total No. of Images** |
| --- | --- | --- | --- | --- | --- |
| **I** | AB2, AB7, M16 | 9 | 4195 | 271-308 | 2667 |
| **II-is** | AB2, AB7, M16 | 9 | 4048 | 262-300 | 2574 |
| **II-ni** | AB2, AB7, M16 | 9 | 3984 | 244-320 | 2533 |
| **III** | AB2, AB7, M16 | 9 | 3990 | 254-293 | 2537 |
| **Va/b** | AB2, AB7, M16 | 9 | 4051 | 270-311 | 2576 |
| **Vc** | AB2, AB7, M16 | 9 | 4095 | 229-302 | 2557 |
| **VI** | AB2, AB7, M16 | 9 | 4113 | 271-319 | 2615 |
| **I-VI** | - | 63 | 28,476 | - | 18,059 |

**Supplementary File 1c. Summary of the stack details obtained from the multiple sampling from all layers of MEC.** 3 stacks of images were acquired in each MEC layer, per case (9 stacks of images in total per layer). The last row shows the sum of all the MEC layers from all cases.

| **Layer** | **Case** | **No. of AS** | **No. of SS** | **No. all synapses** | **% AS (mean)** | **% SS (mean)** | **CFs volume (μm^3^)** | **No. AS**  **/µm^3^ (mean±SD)** | **No. SS**  **/µm^3^ (mean±SD)** | **No. all synapses/µm^3^ (mean±SD)** | **Area of SAS AS (nm^2^; mean ± SE)** | **Area of SAS SS (nm^2^; mean ± SE)** | **Intersynaptic distance (nm; mean±SD)** |
| --- | --- | --- | --- | --- | --- | --- | --- | --- | --- | --- | --- | --- | --- |
| **I** | **AB2** | 422 | 31 | 453 | 93 | 7 | 825 (1,073) | 0.46±0.07 (0.39±0.07) | 0.03±0.01 (0.03±0.00) | 0.49±0.07 (0.42±0.06) | 111,057±6,462 (103,283±6,010) | 46,632±3,449 (43,368±3,208) | 871±84 (844±81) |
|  | **AB7** | 424 | 23 | 447 | 94.9 | 5.1 | 905 (1,117) | 0.42±0.01 (0.38±0.02) | 0.02±001 (0.02±0.00) | 0.44±0.01 (0.40±0.02) | 111,785±5,563 (93,730±5,174) | 55,767±2,562 (51,864±2,383) | 872±61 (846±59) |
|  | **M16** | 419 | 26 | 445 | 94.1 | 5.9 | 844 (1,074) | 0.45±0.08 (0.39±0.06) | 0.03±0.01 (0.02±0.01) | 0.47±0.08 (0.41±0.06) | 105,134±7,055 (97,774±6,561) | 57,701±3,480 (53,662±3,237) | 806±37 (782±36) |
| **II-is** | **AB2** | 423 | 37 | 460 | 91.9 | 8.1 | 1089 (1,122) | 0.35±0.02 (0.38±0.02) | 0.03±0.00 (0.03±0.01) | 0.38±0.01 (0.41±0.01) | 140,997±4,257 (131,127±3,959) | 79,196±6,205 (73,652±5,771) | 917±99 (890±96) |
|  | **AB7** | 427 | 30 | 457 | 93.4 | 6.6 | 951 (974) | 0.40±0.06 (0.44±0.07) | 0.03±0.01 (0.03±0.01) | 0.43±0.06 (0.47±0.07) | 101,566±3,152 (94,456±2,932 | 53,705±3,259 (49,946±3,031) | 868±43 (842±42) |
|  | **M16** | 402 | 33 | 435 | 92.5 | 7.5 | 909 (1,045) | 0.40±0.02 (0.38±0.02) | 0.03±0.01 (0.03±0.01) | 0.43±0.03 (0.42±0.04) | 112,125±4,434 (104,276±4,124) | 71,862±12,206 (66,832±11,351) | 843±49 (817±47) |
| **II-ni** | **AB2** | 428 | 26 | 454 | 94.4 | 5.6 | 927 (988) | 0.42±0.03 (0.43±0.04 | 0.02±0.01 (0.02±0.01) | 0.44±0.05 (0.46±0.05) | 124,176±7,895 (115,484±7,342) | 77,432±12,221 (72,012±11,366) | 846±19 (821±18) |
|  | **AB7** | 483 | 26 | 509 | 95.2 | 4.8 | 1023 (1,083) | 0.42±0.09 (0.45±0.09) | 0.02±0.01 (0.03±0.01) | 0.45±0.10 (0.47±0.11) | 104,123±8,321 (96,834±7,739) | 59,852±12,299 (55,663±11,438) | 827±90 (802±88) |
|  | **M16** | 379 | 45 | 424 | 89.5 | 10.5 | 870 (947) | 0.39±0.01 (0.40±0.02) | 0.04±0.01 (0.05±0.01) | 0.44±0.01 (0.45±0.01) | 127,715±9,094 (118,775±8,458) | 82,140±4,689 (76,391±4,360) | 861±38 (835±37) |
| **III** | **AB2** | 390 | 21 | 411 | 94.8 | 5.2 | 961 (995) | 0.36±0.08 (0.39±0.07) | 0.02±0.01 (0.02±0.01) | 0.38±0.08 (0.41±0.07) | 138,360±10 187 (128,675±9,474) | 97,730±11,236 (90,889±10,449) | 919±147 (891±143) |
|  | **AB7** | 415 | 21 | 436 | 95.3 | 4.7 | 958 (999) | 0.39±0.06 (0.41±0.07) | 0.02±0.01 (0.02±0.01) | 0.41±0.06 (0.44±0.06) | 124,549±11,540 (115,830±10,732) | 51,655±7,341 (48,039±6,827) | 875±65 (849±63) |
|  | **M16** | 411 | 32 | 443 | 92,3 | 7.7 | 982 (1,019) | 0.38±0.11 (0.40±0.11) | 0.03±0.01 (0.03±0.01) | 0.41±0.11 (0.43±0.11) | 128,981±10,056 (119,952±9,352) | 73,365±16,872 (68,229±15,691) | 853±54 (828±52) |
|  |  |  |  |  |  |  |  |  |  |  |  |  |  |
|  |  |  |  |  |  |  |  |  |  |  |  |  |  |
| **Va/b** | **AB2** | 418 | 18 | 436 | 95.8 | 4.2 | 1051 (1,083) | 0.36±0.03 (0.39±0.03) | 0.02±0.006 (0.02±0.006) | 0.37±0.02 (0.40±0.03) | 149,010±2,110 (138,579±1,963) | 59,037±5,827 (54,904±5,419) | 908±46 (881±45) |
|  | **AB7** | 414 | 17 | 431 | 96 | 4 | 1034 (1,059) | 0.36±0.05 (0.39±0.05) | 0.01±0.006 (0.01±0.006) | 0.37±0.06 (0.40±0.06) | 118,640±7,720 (110,335±7,180) | 76,658±14,128 (71,292±13,139) | 866±49 (840±47) |
|  | **M16** | 313 | 15 | 328 | 95.5 | 4.5 | 932 (988) | 0.30±0.05 (0.31±0.05) | 0.01±0.003 (0.01±0.003) | 0.31±0.05 (0.33±0.06) | 112,292±21,735 (104,431±20,214) | 88,399±17,309 (82,211±16,098) | 921±44 (894±43) |
| **Vc** | **AB2** | 475 | 23 | 498 | 95.4 | 4.6 | 1028 (1,056) | 0.41±0.06 (0.45±0.7) | 0.02±0.01 (0.02±0.01) | 0.44±0.06 (0.47±0.06) | 127,546±6,938 (118,618±6,453) | 82,215±4,379 (76,460±4,073) | 854±53 (828±51) |
|  | **AB7** | 413 | 31 | 444 | 92.9 | 7.9 | 940 (963) | 0.39±0.05 (0.43±0.05) | 0.03±0.00 (0.03±0.00) | 0.42±0.05 (0.46±0.05) | 122,849±11,604 (114,249±10,792) | 85,620±9,319 (79,626±8,666) | 899±71 (872±69) |
|  | **M16** | 369 | 33 | 402 | 92 | 8 | 1016 (1,099) | 0.33±0.11 (0.33±0.12) | 0.03±0.01 (0.03±0.01) | 0.35±0.12 (0.37±0.13) | 128,612±10,535 (119,474±9,797) | 94,297±9,401 (87,697±8,743) | 845±83 (819±80) |
| **VI** | **AB2** | 409 | 11 | 420 | 97.4 | 3.6 | 1020 (1,075) | 0.36±0.03 (0.38±0.03) | 0.01±0.00 (0.01±0.00) | 0.37±0.03 (0.39±0.03) | 110,144±8,402 (102,434±7,814) | 85,517±21,764 (79,531±20,241) | 861±71 (835±69) |
|  | **AB7** | 361 | 17 | 378 | 95.2 | 4.8 | 1107 (1,130) | 0.29±0.04 (0.32±0.05) | 0.01±0.01 (0.02±0.01) | 0.31±0.04 (0.33±0.04) | 77,022±4,344 (71,630±4,040) | 38,043±10,184 (35,380±9,472) | 897±68 (870±66) |
|  | **M16** | 407 | 25 | 432 | 94.2 | 5.8 | 992 (1,035) | 0.37±0.06 (0.40±0.07) | 0.02±0.01 (0.02±0.01) | 0.39±0.06 (0.42±0.07) | 111,303±5,379 (103,511±5,002) | 87,858±5,964 (81,708±5,547) | 796±79 (772±75) |

Supplementary File 1d. Accumulated data acquired from the ultrastructural analysis of neuropil from layers I, II-is, II-ni, III, Va/b, Vc and VI of the MEC, for individual cases. Data in parentheses are not corrected for shrinkage. AS: asymmetric synapses; CF: counting frame; SD: standard deviation; SE: standard error of the mean; SS: symmetric synapses.

|  | **AS** | | | **SS** | | |
| --- | --- | --- | --- | --- | --- | --- |
|  | ***n*** | **Μ** | **σ** | ***n*** | **μ** | **σ** |
| **Layer I** | 1,253 | 11.34 | 0.71 | 80 | 10.65 | 0.71 |
| **Layer II-is** | 1,255 | 11.44 | 0.74 | 100 | 11.01 | 0.55 |
| **Layer II-ni** | 1,290 | 11.34 | 0.70 | 97 | 11.03 | 0.55 |
| **Layer III** | 1,210 | 11.48 | 0.78 | 74 | 10.97 | 0.75 |
| **Layer Va/b** | 1,143 | 11.60 | 0.69 | 50 | 11.04 | 0.61 |
| **Layer Vc** | 1,444 | 11.40 | 0.67 | 96 | 11.08 | 0.46 |
| **Layer VI** | 1,175 | 11.26 | 0.75 | 53 | 10.92 | 0.82 |
| **Layers I–VI** | 8,770 | 11.44 | 0.72 | 550 | 10.97 | 0.64 |

Supplementary File 1e. Number of synaptic SAS analyzed (n), the location (µ) and scale (σ) of the best-fit log-normal distributions in the six cortical layers. AS: asymmetric synapses; SAS: synaptic apposition surface; SS: symmetric synapses

| **Layer** | **Type of Synapse** | **Macular** | **Perforated** | **Horseshoe-shaped** | **Fragmented** | **Complex** |
| --- | --- | --- | --- | --- | --- | --- |
| **I** | **AS** | 87% (1100) | 11.4% (143) | 1.3% (17) | 0.3% (4) | 13% (164) |
|  | **SS** | 77.5% (62) | 15.1% (12) | 1.2% (1) | 6.2% (5) | 22.5% (18) |
| **II-is** | **AS** | 88.2% (1102) | 9% (113) | 2.2% (27) | 0.6% (8) | 11.8% (148) |
|  | **SS** | 86.9% (86) | 8.1% (8) | 3% (3) | 2% (2) | 13.1% (13) |
| **II-ni** | **AS** | 86.3% (1110) | 11% (142) | 2.1% (27) | 0.6% (8) | 13.7% (177) |
|  | **SS** | 73.2% (71) | 18.6% (18) | 7.2% (7) | 1% (1) | 26.8% (26) |
| **III** | **AS** | 78.9% (956) | 17.4% (211) | 2.7% (32) | 1% (12) | 21.1% (255) |
|  | **SS** | 74.3% (55) | 18.9% (14) | 2.7% (2) | 4.1% (3) | 25.7% (19) |
| **Va/b** | **AS** | 81.6% (933) | 14.6% (167) | 2.6% (30) | 1.2% (14) | 18.4% (211) |
|  | **SS** | 74% (37) | 14% (7) | 4% (2) | 8% (4) | 26% (13) |
| **Vc** | **AS** | 86.6% (1081) | 8.8% (110) | 3% (37) | 1.6% (20) | 13.4% (167) |
|  | **SS** | 81.2% (69) | 11.8% (10) | 3.5% (3) | 3.5% (3) | 18.8% (16) |
| **VI** | **AS** | 77.6% (913) | 15.4% (181) | 3.8% (45) | 3.2% (37) | 22.4% (263) |
|  | **SS** | 59.3% (32) | 25.9% (14) | 7.4% (4) | 7.4% (4) | 40.7% (22) |
| **I-VI** | **AS** | 83.9% (7195) | 12.4% (1067) | 2.5% (215) | 1.2% (103) | 16.1% (1.385) |
|  | **SS** | 76.4% (412) | 15.4% (83) | 4.1% (22) | 4.1% (22) | 23.6% (127) |

Supplementary File 1f. Proportion of the different shapes of synaptic junctions in MEC layers. Data in parentheses refer to absolute numbers of synapses.

| **Layer** | **Case** | **Type of Synapse** | **Macular** | **Perforated** | **Horseshoe-shaped** | **Fragmented** | **Complex** |
| --- | --- | --- | --- | --- | --- | --- | --- |
| **I** | **AB2** | **AS** | 88.4% (373) | 10.2% (43) | 0.9% (4) | 0.5% (2) | 11.6% (49) |
|  |  | **SS** | 74.2% (23) | 16.1% (5) | - (0) | 9.7% (3) | 25.8% (8) |
|  | **AB7** | **AS** | 89.6% (379) | 8.8% (37) | 1.6% (7) | - (0) | 10.4% (44) |
|  |  | **SS** | 78.2% (18) | 13% (3) | 4.4% (1) | 4.4% (1) | 21.8% (5) |
|  | **M16** | **AS** | 83.1% (348) | 15% (63) | 1.4% (6) | 0.5% (2) | 16.9% (71) |
|  |  | **SS** | 80.8% (21) | 15.4% (4) | - (0) | 3.8% (1) | 19.2% (5) |
|  | **Total** | **AS** | 87% (1100) | 11.4% (143) | 1.3% (17) | 0.3% (4) | 13% (164) |
|  |  | **SS** | 77.5% (62) | 15.1% (12) | 1.2% (1) | 6.2% (5) | 22.5% (18) |
| **II-is** | **AB2** | **AS** | 92.7% (392) | 6.2% (26) | 0.9% (4) | 0.2% (1) | 8.3% (31) |
|  |  | **SS** | 86.5% (32) | 8.1% (3) | 2.7% (1) | 2.7% (1) | 13.5% (5) |
|  | **AB7** | **AS** | 90.4% (386) | 6.1% (26) | 2.8% (12) | 0.7% (3) | 9.6% (41) |
|  |  | **SS** | 90% (27) | 6.7% (2) | - (0) | 3.3% (1) | 10% (3) |
|  | **M16** | **AS** | 81% (324) | 15.2% (61) | 2.8% (11) | 1% (4) | 19% (76) |
|  |  | **SS** | 84.4% (27) | 9.3% (3) | 6.3% (2) | - (0) | 15.6% (5) |
|  | **Total** | **AS** | 88.2% (1102) | 9% (113) | 2.2% (27) | 0.6% (8) | 11.8% (148) |
|  |  | **SS** | 86.9% (86) | 8.1% (8) | 3% (3) | 2% (2) | 13.1% (13) |
| **II-ni** | **AB2** | **AS** | 91.3% (390) | 7.3% (31) | 1.2% (5) | 0.2% (1) | 8.7% (37) |
|  |  | **SS** | 84.6% (22) | 3.8% (1) | 7.8% (2) | 3.8% (1) | 15.4% (4) |
|  | **AB7** | **AS** | 86.5% (418) | 10.4% (50) | 2.5% (12) | 0.6% (3) | 13.5% (65) |
|  |  | **SS** | 73.1% (19) | 23.1% (6) | 3.8% (1) | - (0) | 26.9% (7) |
|  | **M16** | **AS** | 80.1% (302) | 16.1% (61) | 2.7% (10) | 1.1% (4) | 19.9% (75) |
|  |  | **SS** | 66.7% (30) | 24.4% (11) | 8.9% (4) | - (0) | 33.3% (15) |
|  | **Total** | **AS** | 86.3% (1110) | 11% (142) | 2.1% (27) | 0.6% (8) | 13.7% (177) |
|  |  | **SS** | 73.2% (71) | 18.6% (18) | 7.2% (7) | 1% (1) | 26.8% (26) |
| **III** | **AB2** | **AS** | 80.8% (311) | 16.0% (62) | 1.6% (6) | 1.6% (6) | 19.2% (74) |
|  |  | **SS** | 71.4% (15) | 28.6% (6) | - (0) | - (0) | 28.6% (6) |
|  | **AB7** | **AS** | 80.7% (335) | 14.9% (62) | 3.7% (15) | 0.7% (3) | 19.3% (80) |
|  |  | **SS** | 85.7% (18) | 4.8% (1) | - (0) | 9.5% (2) | 14.3% (3) |
|  | **M16** | **AS** | 75.4% (310) | 21.2% (87) | 2.7% (11) | 0.7% (3) | 24.6% (101) |
|  |  | **SS** | 68.8% (22) | 21.9% (7) | 6.2% (2) | 3.1% (1) | 31.2% (10) |
|  | **Total** | **AS** | 78.9% (956) | 17.4% (211) | 2.7% (32) | 1% (12) | 21.1% (255) |
|  |  | **SS** | 74.3% (55) | 18.9% (14) | 2.7% (2) | 4.1% (3) | 25.7% (19) |
| **Va/b** | **AB2** | **AS** | 79.7% (333) | 18.1% (76) | 1% (4) | 1.2% (5) | 20.3% (85) |
|  |  | **SS** | 55.6% (10) | 33.2% (6) | 5.6% (1) | 5.6% (1) | 44.4% (8) |
|  | **AB7** | **AS** | 86.4% (357) | 9.4% (39) | 2.8% (12) | 1.2% (5) | 13.6% (56) |
|  |  | **SS** | 82.4% (14) | - (0) | - (0) | 17.6% (3) | 17.6% (3) |
|  | **M16** | **AS** | 77.6% (243) | 16.6% (52) | 4.5% (14) | 1.3% (4) | 22.4% (70) |
|  |  | **SS** | 86.6% (13) | 6.7% (1) | 6.7% (1) | - (0) | 13.4% (2) |
|  | **Total** | **AS** | 81.6% (933) | 14.6% (167) | 2.6% (30) | 1.2% (14) | 18.4% (211) |
|  |  | **SS** | 74% (37) | 14% (7) | 4% (2) | 8% (4) | 26% (13) |
| **Vc** | **AB2** | **AS** | 87.4% (415) | 8.6% (41) | 2.3% (11) | 1.7% (8) | 12.6% (60) |
|  |  | **SS** | 69.6% (16) | 26.1% (6) | 4.3% (1) | - (0) | 30.4% (7) |
|  | **AB7** | **AS** | 89.8% (368) | 6.6% (27) | 2.6% (11) | 1% (4) | 10.2% (42) |
|  |  | **SS** | 93.5% (29) | - (0) | 6.5% (2) | - (0) | 6.5% (2) |
|  | **M16** | **AS** | 82.1% (298) | 11.6% (42) | 4.1% (15) | 2.2% (8) | 17.9% (65) |
|  |  | **SS** | 77.4% (24) | 12.9% (4) | - (0) | 9.7% (3) | 22.6% (7) |
|  | **Total** | **AS** | 86.6% (1081) | 8.8% (110) | 3% (37) | 1.6% (20) | 13.4% (167) |
|  |  | **SS** | 81.2% (69) | 11.8% (10) | 3.5% (3) | 3.5% (3) | 18.8% (16) |
| **VI** | **AB2** | **AS** | 80.6% (329) | 11.5% (47) | 4.7% (19) | 3.2% (13) | 19.4% (79) |
|  |  | **SS** | 50% (6) | 33.3% (4) | 16.7% (2) | - (0) | 50% (6) |
|  | **AB7** | **AS** | 77.6% (280) | 18% (65) | 2.7% (10) | 1.7% (6) | 22.4% (81) |
|  |  | **SS** | 64.7% (11) | 23.5% (4) | 11.8% (2) | - (0) | 35.3% (6) |
|  | **M16** | **AS** | 74.7% (304) | 17% (69) | 3.9% (16) | 4.4% (18) | 25.3% (103) |
|  |  | **SS** | 60% (15) | 24% (6) | - (0) | 16% (4) | 40% (10) |
|  | **Total** | **AS** | 77.6% (913) | 15.4% (181) | 3.8% (45) | 3.2% (37) | 22.4% (263) |
|  |  | **SS** | 59.3% (32) | 25.9% (14) | 7.4% (4) | 7.4% (4) | 40.7% (22) |
| **I-VI** | **AB2** | **AS** | 86% (2543) | 11% (326) | 1.8% (53) | 1.2% (36) | 14% (415) |
|  |  | **SS** | 73.8% (124) | 18.5% (31) | 4.2% (7) | 3.6% (6) | 26.2% (44) |
|  | **AB7** | **AS** | 86.1% (2523) | 10.4% (306) | 2.7% (79) | 0.8% (24) | 13.9% (409) |
|  |  | **SS** | 82.4% (136) | 9.7% (16) | 3.7% (6) | 4.2% (7) | 17.6% (29) |
|  | **M16** | **AS** | 79.1% (2129) | 16.2% (435) | 3.1% (83) | 1.6% (43) | 20.9% (561) |
|  |  | **SS** | 73.7% (152) | 17.5% (36) | 4.4% (9) | 4.4% (9) | 26.3% (54) |
|  | **Total** | **AS** | 83.9% (7195) | 12.4% (1067) | 2.5% (215) | 1.2% (103) | 16.1% (1385) |
|  |  | **SS** | 76.4% (412) | 15.4% (83) | 4.1% (22) | 4.1% (22) | 23.6% (127) |

Supplementary File 1g. Proportion of the different shapes of synaptic junctions in MEC layers, for individual cases. Data in parentheses refer to absolute numbers of synapses.

| **Layer** | **Type of Synapse** | **Macular** | **Perforated** | **Horseshoe-Shaped** | **Fragmented** | **Complex** |
| --- | --- | --- | --- | --- | --- | --- |
| **I** | **AS** | 92,661 ± 3,095 (86,175 ± 2,878) | 197,654 ± 16,398 (183,819 ± 15,250) | 250,911 ± 40,834 (23,348 ± 37,975) | 267,410 ± 81,563 (248,691 ± 75,853) | 207,129 ± 20,454 (192,630 ±19,023) |
|  | **SS** | 46,580 ± 4,067 (43,319 ± 3,782) | 79,066 ± 13,079 (73,531 ± 12,163) | 52,190 (48,537) | 66,841 ± 25,203 (62,163 ± 23,439) | 80,379 ± 14,474 (74,752 ± 13,461) |
| **II-is** | **AS** | 101,988 ± 7,669 (94,849 ± 7,133) | 247,884 ± 12,526 (230,532 ± 11,649) | 221,102 ± 35,576 (205,625 ± 33,086) | 206,647 ± 34,173 (192,182 ± 31,781) | 237,901 ± 12,971 (221,239 ± 12,065) |
|  | **SS** | 65,361 ± 5,228 (60,785 ± 4,863) | 76,540 ± 22,208 (71,182 ± 20,653) | 61,474 ± 11,105 (57,171 ± 10,328) | 80,562 ± 17,663 (74,922 ± 16,426) | 72,531 ± 10,711 (67,454 ± 9,961) |
| **II-ni** | **AS** | 102,985 ± 4,837 (95,776 ± 4,499) | 208,141 ± 11,035 (193,571 ± 10,263) | 239,286 ± 20,749 (222,536 ± 19,297) | 210,844 ± 35,973 (196,085 ± 33,455) | 211,689 ± 10,498 (196,871 ± 9,763) |
|  | **SS** | 63,792 ± 7,004 (59,327 ± 6,514) | 75,745 ± 17,776 (70,443 ± 16,531) | 90,677 ± 13,656 (84,330 ± 12,700) | 38,487 (35,793) | 81,962 ± 11,991 (76,224 ± 11,152) |
| **III** | **AS** | 98,632 ± 5,098 (91,728 ± 4,741) | 244,457 ± 10,273 (227,345 ± 9,554) | 221,461 ± 27,813 (205,958 ± 25,866) | 256,773 ± 29,314 (238,799 ± 27,262) | 244,603 ± 10,271 (227,481 ± 9,552) |
|  | **SS** | 68,073 ± 9,960 (63,308 ± 9,263) | 89,039 ± 9,893 (82,806 ± 9,200) | 87,262 ± 43,491 (81,154 ± 40,447) | 150,035 ± 78,735 (139,532 ± 73,223) | 90,822 ± 11,898 (84,465 ±11,065) |
| **Va/b** | **AS** | 112,898 ± 4,214 (104,995 ± 3,919) | 242,129 ± 11,233 (225,180 ± 10,447) | 220,571 ± 11,053 (205,131 ± 10,279) | 253,021 ± 32,899 (235,310 ± 30,596) | 238,420 ± 9,232 (221,731 ± 8,586) |
|  | **SS** | 65,199 ± 10,194 (60,635 ± 9,481) | 82,702 ± 8,009 (76,913 ± 7,448) | 40,590 ± 886 (37,749± 824) | 122,609 ± 15,686 (114,026 ± 14,588) | 91,303 ± 14,198 (84,911 ± 13,204) |
| **Vc** | **AS** | 108,431 ± 5,220 (100,841 ± 4,854) | 240,291 ± 17,494 (223,470 ± 16,269) | 243,754 ± 25,012 (226,691 ± 23,262) | 265,690 ± 24,100 (247,092 ± 22,413) | 108,431 ± 5,220 (100,841 ± 4,854) |
|  | **SS** | 83,176 ± 5,150 (77,354 ± 4,790) | 111,784 ± 13,144 (103,959 ± 12,224) | 73,753 ± 17,089 (68,590 ± 15,892) | 101,698 ± 15,405 (94,580 ± 14,327) | 83,176 ± 5,150 (77,354 ± 4,790) |
| **VI** | **AS** | 79,331 ± 4,767 (73,778 ± 4,433) | 168,558 ± 15,001 (156,759 ± 13,951) | 183,334 ± 14,939 (170,501 ± 13,893) | 191,595 ± 19,309 (178,183 ± 17,957) | 170,959 ± 12,388 (158,992 ± 11,521) |
|  | **SS** | 44,170 ± 6,484 (41,078 ± 6,030) | 119,142 ± 13,275 (110,802 ± 12,346) | 62,251 ± 17,584 (57,894 ± 16,354) | 129,831 ± 35,440 (120,742 ± 32,959) | 110,547 ± 13,584 (102,808 ± 12,634) |
| **I-VI** | **AS** | 99,561 ± 2,260 (92,592 ± 2,101) | 221,302 ± 6,078 (205,811 ± 5,653) | 225,369 ± 9,858 (209,593 ± 9,168) | 233,668 ± 11,949 (217,311 ± 11,112) | 99,561 ± 2,260 (92,592 ± 2,101) |
|  | **SS** | 62,336 ± 3,029 (57,972 ± 2,817) | 91,444 ± 5,739 (85,043 ± 5,337) | 70,680 ± 7,324 (65,733 ± 6,811) | 102,349 ± 15,452 (95,185 ± 14,371) | 62,336 ± 3,029 (57,972 ± 2,817) |

Supplementary File 1h. SAS area of asymmetric (AS) and symmetric (SS) synapses for each synaptic shape, in each MEC layer. Data in parentheses are not corrected for shrinkage. Values (in nm^2^) are expressed as mean ± standard error.

| **Layer** | **Case** | **Tipe of Synapse** | **Macular** | **Perforated** | **Horseshoe** | **Fragmented** | **Complex** |
| --- | --- | --- | --- | --- | --- | --- | --- |
| **I** | **AB2** | **AS** | 101,222 ± 3,039 (94,136 ± 2,827) | 184,880 ± 29,944 (171,938 ± 27,848) | 175,065 ± 59,004 (162,810 ± 54,874) | 322,942 ± 103,471 (300,036 ± 96,228) | 191,851 ± 34,956 (178,422 ± 32,509) |
|  |  | **SS** | 38,842 ± 2,120 (36,123 ± 1,972) | 59,168 ± 5,204 (55,026 ± 4,840) | - | 85,613 (79,620) | 90,854 ± 36,558 (84,494 ± 33,999) |
|  | **AB7** | **AS** | 89,966 ± 3,225 (83,669 ± 2,999) | 212,981 ± 44,328 (198,072 ± 41,225) | 348,624 ± 72,012 (324,220 ± 66,971) | - | 235,190 ± 54,498 (218,727 ± 50,683) |
|  |  | **SS** | 55,430 ± 1,433 (51,550 ± 1,333) | 77,980 ± 27,699 (72,521 ± 25,760) | 52,190 (48,537) | 36,352 (33,808) | 66,743 ± 19,545 (62,071 ± 18,177) |
|  | **M16** | **AS** | 86,795 ± 6,152 (80,720 ± 5,721) | 195,103 ± 12,753 (181,446 ± 11,860) | 203,763 ± 37,594 (189,500 ± 34,962) | 156,347 (145,403) | 194,345 ± 15,380 (180,741 ± 14,303) |
|  |  | **SS** | 45,467 ± 11,054 (42,285 ± 10,280) | 100,051 ± 30,562 (93,048 ± 28,423) | - | 41,015 (38,144) | 85,118 ± 15,629 (79,160 ± 14,535) |
| **II-is** | **AB2** | **AS** | 130,221 ± 5,857 (121,105 ± 5,447) | 286,237 ± 21,844 (266,201 ± 20,315) | 300,127 ± 94,855 (279,118 ± 88,215) | 175,889 (163,577) | 278,763 ± 26,510 (259,250 ± 24,655) |
|  |  | **SS** | 73,872 ± 7,156 (68,701 ± 6,655) | 141,358 (131,463) | 50,369 (46,844) | 62,899 (58,496) | 86,056 ± 35,687 (80,032 ± 33,189) |
|  | **AB7** | **AS** | 89,423 ± 4,109 (83,164 ± 3,821) | 232,829 ± 15,903 (216,531 ± 14,790) | 183,706 ± 37,076 (170,847 ± 34,480) | 166,100 ± 36,220 (154,473 ± 33,685) | 216,045 ± 6,865 (200,922 ± 6,385) |
|  |  | **SS** | 53,519 ± 3,588 (49,773 ± 3,337) | 40,445 (37,614) | - | 98,224 (91,348) | 69,335 ± 28,890 (64,481 ± 26,867) |
|  | **M16** | **AS** | 86,321 ± 7,379 (80,279 ± 6,863) | 224,586 ± 5,709 (208,865 ± 5,309) | 179,473 ± 11,288 (166,910 ± 10,498) | 282,845 ± 69,543 (263,046 ± 64,675) | 218,864 ± 3,840 (203,544 ± 3,571) |
|  |  | **SS** | 68,691 ± 12,332 (63,882 ± 11,469) | 62,178 ± 868 (58,095 ± 808) | 72,579 (67,499) | - | 65,645 ± 3,503 (61,050 ± 3,258) |
| **II-ni** | **AB2** | **AS** | 114,835 ± 6,400 (106,796 ± 5,952) | 211,495 ± 24,500 (196,690 ± 22,785) | 269,605 ± 15,871 (250,733 ± 56,240) | 273,833 (254,665) | 222,354 ± 29,133 (205,118 ± 27,093) |
|  |  | **SS** | 75,232 ± 13,131 (69,966 ± 12,211) | 45,204 (42,040) | 117,137 (108,937) | 38,487 (35,793) | 65,823 ± 27,336 (61,215 ± 25,423) |
|  | **AB7** | **AS** | 90,025 ± 8,873 (83,723 ± 8,252) | 189,558 ± 17,745 176,289 (16,503) | 224,039 ± 9,971 (208,356 ± 9,273) | 281,632 ± 46,258 (261,918 ± 43,020) | 196,410 ± 10,908 (179,157 ± 10,144) |
|  |  | **SS** | 41,902 ± 4,454 (38,969 ± 4,143) | 74,983 ± 30,956 (69,734 ± 28,790) | 71,960 (66,923) | - | 79,189 ± 28,356 (73,646 ± 26,371) |
|  | **M16** | **AS** | 104,096 ± 2,504 (96,809 ± 2,329) | 223,371 ± 15,871 (207,735 ± 16,503) | 224,214 ± 26,844 (208,519 ± 24,965) | 142,656 ± 33,174 (132,670 ± 30,852) | 216,305 ± 13,061 (208,541 ± 12,146) |
|  |  | **SS** | 74,241 ± 6072 (69,044 ± 5,647) | 86,688 ± 32,217 (80,620 ± 29,962) | 86,806 ± 24,058 (80,730 ± 22,374) | - | 95,494 ± 10,325 (88,809 ± 9,602) |
| **III** | **AB2** | **AS** | 110,929 ± 7,912 (103,164 ± 7,358) | 247,376 ± 8,401 (230,059 ± 7,813) | 257,345 ± 62,238 (239,331 ± 57,881) | 295,531 ± 37,375 (274,844 ± 34,759) | 253,575 ± 4,765 (235,825 ± 4,431) |
|  |  | **SS** | 101,194 ± 14,339 (94,111 ± 13,335) | 82,440 ± 3,302 (76,669 ± 2,820) | - | - | 82,440 ± 3,032 (76,669 ± 2,820) |
|  | **AB7** | **AS** | 93,165 ± 6,029 (86,644 ± 5,607) | 250,210 ± 32,209 (232,695 ± 9,954) | 246,375 ± 30,086 (229,129 ± 27,980) | 157,049 ± 26,305 (146,055 ± 24,463) | 247,938 ± 32,422 (230,582 ± 30,152) |
|  |  | **SS** | 49,072 ± 10,065 (45,637 ± 9,361) | 45,371 (42,195) | - | 71,687 ± 13,513 (66,669 ± 12,568) | 61,730 ± 3,556 (57,409 ± 3,307) |
|  | **M16** | **AS** | 91,802 ± 9,950 (85,376 ± 9,254) | 235,785 ± 10,000 (219,280 ± 9,300) | 160,662 ± 41,252 (149,415 ± 38,364) | 284,496 ± 44,073 (264,582 ± 40,988) | 232,297 ± 8,405 (216,037 ± 7,817) |
|  |  | **SS** | 53,953 ± 7,397 (50,176 ± 6,880) | 110,193 ± 10,607 (102,480 ± 9,865) | 87,262 ± 43,491 (81,154 ± 40,447) | 306,729 (285,258) | 118,599 ± 24,592 (110,297 ± 22,871) |
| **Va/b** | **AB2** | **AS** | 123,697 ± 5,048 (115,038 ± 4,695) | 236,015 ± 13,588 (219,494 ± 12,637 | 241,207 ± 28,095 (224,323 ± 26,128) | 325,348 ± 8,295 (302,574 ± 7,714) | 242,546 ± 9,149 (225,568 ± 8,509) |
|  |  | **SS** | 38,564 ± 13,183 (35,865 ± 12,260) | 77,504 ± 8,616 (72,078 ± 8,013) | 39,704 (36,925) | 92,963 (86,456) | 74,827 ± 10,849 (69,589 ± 10,090) |
|  | **AB7** | **AS** | 100,378 ± 4,258 (93,351 ± 3,960) | 234,281 ± 14,117 (217,882 ± 12,637) | 207,968 ± 15,552 (193,410 ± 14,463) | 238,907 ± 69,200 (222,183 ± 64,356) | 229,614 ± 14,206 (213,541 ± 13,211) |
|  |  | **SS** | 67,421 ± 9,282 (62,702 ± 8,632) | - | - | 137,432 ± 8,890 (127,812 ± 8,268) | 137,432 ± 8,890 (127,812 ± 8,268) |
|  | **M16** | **AS** | 114,620 ± 5,597 (106,596 ± 5,205) | 256,091 ± 31,359 (238,165 ± 29,164) | 212,538 ± 10,497 (197,661 ± 9,762) | 158,645 ± 27,959 (147,540 ± 26,002) | 243,100 ± 26,059 (226,083 ± 24,235) |
|  |  | **SS** | 89,611 ± 18,220 (83,338 ± 16,945) | 98,297 (91,416) | 41,476 (38,573) | - | 69,887 ± 28,410 (64,995 ± 26,421) |
| **Vc** | **AB2** | **AS** | 108,768 ± 2,541 (101,155 ± 2,363) | 245,585 ± 22,685 (228,394 ± 21,097) | 302,117 ± 48,067 (280,969 ± 44,703) | 250,032 ± 32,883 (232,530 ± 30,581) | 250,483 ± 23,052 (225,568 ± 8,509) |
|  |  | **SS** | 71,875 ± 811 (66,844 ± 754) | 117,148 ± 6,131 (108,948 ± 5,702) | 74,859 (69,619) | - | 111,096 ± 78 (103,310 ± 73) |
|  | **AB7** | **AS** | 111,861 ± 11,060 (104,031 ± 10,286) | 221,124 ± 5,818 (205,645 ± 5,411) | 221,937 ± 41,817 (206,401 ± 38,890) | 231,859 ± 79,721 (215,629 ± 74,141) | 223,918 ± 19,853 (213,541 ± 13,211) |
|  |  | **SS** | 86,082 ± 8,400 (80,056 ± 7,812) | - | 73,200 ± 29,583 (68,076 ± 27,512) | - | 73,200 ± 29,583 (8,076 ± 27,512) |
|  | **M16** | **AS** | 104,665 ± 13,607 (97,338 ± 12,655) | 254,163 ± 53,198 (236,371 ± 49,474) | 207,207 ± 28,972 (192,703 ± 26,944) | 303,903 ± 31,417 (282,630 ± 29,218) | 244,450 ± 39,981 (227,338 ± 37,182) |
|  |  | **SS** | 91,570 ± 11,990 (85,160 ± 11,151) | 108,207 ± 23,396 (100,633 ± 21,758) | - | 101,698 ± 15,405 (94,580 ± 14,327) | 108,844 ± 32,951 (101,225 ± 16,404) |
| **VI** | **AB2** | **AS** | 86,228 ± 6,132 (80,192 ± 5,703) | 220,662 ± 15,119 (205,216 ± 14,060) | 177,145 ± 38,427 (164,745 ± 35,737) | 224,731 ± 42,881 (209,000 ± 39,879) | 209,198 ± 8,633 (194,554 ± 8,029) |
|  |  | **SS** | 39,253 ± 5,372 (36,506 ± 4,996) | 130,164 ± 40,331 (121,053 ± 37,508) | 94,885 (88,243) | - | 131,427 ± 39,068 (122,227 ± 36,333) |
|  | **AB7** | **AS** | 63,184 ± 3,185 (58,761 ± 2,962) | 121,789 ± 2,559 (113,264 ± 2,380) | 165,155 ± 26,120 (153,594 ± 24,291) | 144,688 ± 27,602 (134,560 ± 25,669) | 126,350 ± 3,346 (117,506 ± 3,111) |
|  |  | **SS** | 28,110 ± 5,038 (26,143 ± 4,686) | 90,385 ± 9,873 (84,058 ± 9,182) | 45,935 ± 11,354 (42,719 ± 10,560) | - | 71,063 ± 3,643 (66,088 ± 3,388) |
|  | **M16** | **AS** | 88,482 ± 5,290 (82, 382 ± 4,920) | 163,224 ± 185 (151,798 ± 173) | 207,702 ± 6,165 (193,163 ± 5,734) | 205,366 ± 11,372 (190,990 ± 10,576) | 177,328 ± 3,086 (164,915 ± 2,870) |
|  |  | **SS** | 65,146 ± 9,449 (60,586 ± 8,787) | 130,965 ± 16,519 (121,798 ± 5,362) | - | 129,831 ± 35,440 (120,742 ± 32,959) | 122,949 ± 6,055 (114,342 ± 5,631) |

Supplementary File 1i. SAS area of asymmetric (AS) and symmetric (SS) synapses for each synaptic shape, in each MEC layer, per individual case. Data in parentheses are not corrected for shrinkage. Values (in nm^2^) are expressed as mean ± standard error.

| **Layer** | **Type of Synapse** | **Spine Head** | **Spine Neck** | **Spiny Shaft** | **Aspiny Shaft** | **Shafts**  **(Spiny + Aspiny)** |
| --- | --- | --- | --- | --- | --- | --- |
| **I** | **AS** | 54.2%  (675) | 0.4%  (5) | 17.5%  (218) | 21.9%  (273) | 39.4%  (491) |
|  | **SS** | 0.6%  (7) | 0.2%  (3) | 1.8%  (23) | 3.4%  (43) | 5.2%  (66) |
| **II-is** | **AS** | 57.5%  (740) | 0.6%  (8) | 15.7%  (202) | 18.5%  (240) | 34.2%  (442) |
|  | **SS** | 1.1%  (14) | 0.2%  (2) | 3.9%  (50) | 2.5%  (32) | 6.4%  (82) |
| **II-ni** | **AS** | 59.5%  (798) | 0.2%  (3) | 16.2%  (217) | 17.1%  (229) | 33.3%  (446) |
|  | **SS** | 0.5%  (7) | 0.1%  (2) | 4.2%  (56) | 2.2%  (30) | 6.4%  (86) |
| **III** | **AS** | 54.9%  (667) | 0.5%  (6) | 20.9%  (255) | 17.9%  (218) | 38.8%  (473) |
|  | **SS** | 0.7%  (9) | -  (0) | 3.0%  (37) | 2.1%  (26) | 5.1%  (63) |
| **Va/b** | **AS** | 52.9%  (571) | 1.0%  (11) | 21.5%  (232) | 20.9%  (226) | 42.4%  (458) |
|  | **SS** | 0.1%  (1) | 0.2%  (2) | 1.8%  (19) | 1.6%  (17) | 3.4%  (36) |
| **Vc** | **AS** | 60.5%  (761) | 0.7%  (9) | 14.5%  (183) | 17.8%  (223) | 32.3%  (406) |
|  | **SS** | 1.4%  (18) | 0.1%  (1) | 2.6%  (33) | 2.4%  (30) | 5%  (63) |
| **VI** | **AS** | 76.2%  (850) | 0.3%  (3) | 9.4%  (106) | 9.8%  (109) | 19.2%  (215) |
|  | **SS** | 0.6%  (6) | 0.1%  (1) | 2.2%  (24) | 1.4%  (16) | 3.6%  (40) |
| **I-VI** | **AS** | 59.3%  (5062) | 0.5%  (45) | 16.5%  (1413) | 17.8%  (1518) | 34.3%  (2931) |
|  | **SS** | 0.7%  (62) | 0.1%  (11) | 2.8%  (242) | 2.3%  (194) | 5.1%  (436) |

**Supplementary File 1j. Proportion of the different postsynaptic targets in MEC layers.** Synapses established on spine head include both complete and incomplete spines. Data in parentheses refer to the absolute number of synapses found in each layer.

| **Layer** | **Case** | **Type of Synapse** | **Complete**  **Spine Heads** | **Incomplete Spine Heads** | **Spine Necks** | **Spiny Shaft** | **Aspiny Shaft** | **Shafts**  **(Spiny + Aspiny)** |
| --- | --- | --- | --- | --- | --- | --- | --- | --- |
| **I** | **AB2** | **AS** | 41.6% (160) | 13.0% (50) | 0.3% (1) | 18% (69) | 27.1% (104) | 45.1% (173) |
|  |  | **SS** | - (0) | - (0) | 3.5% (1) | 17.2% (5) | 79.3% (23) | 96.5% (27) |
|  | **AB7** | **AS** | 39.6% (156) | 17.6% (69) | 1% (4) | 17.6% (63) | 24.2% (95) | 41.8% (158) |
|  |  | **SS** | - (0) | 23.8% (5) | 4.8% (1) | 33.3% (7) | 38.1% (8) | 71.4% (15) |
|  | **M16** | **AS** | 40.1% (158) | 20.8% (82) | - (0) | 20.3% (80) | 18.8% (74) | 39.1% (154) |
|  |  | **SS** | 3.8% (1) | 3.8% (1) | 3.8% (1) | 42.4% (11) | 46.2% (12) | 88.6% (23) |
|  | **Total** | **AS** | 40.5% (474) | 17.2% (201) | 0.4% (5) | 18.6% (218) | 23.3% (273) | 41.9% (491) |
|  |  | **SS** | 1.3% (1) | 7.9% (6) | 3.9% (3) | 30.3% (23) | 56.6% (43) | 86.9% (66) |
| **II-is** | **AB2** | **AS** | 38.5% (155) | 17.4% (70) | 0.2% (1) | 14.4% (58) | 29.5% (119) | 43.9% (177) |
|  |  | **SS** | 10.8% (4) | - (0) | 2.7% (1) | 51.4% (19) | 35.1% (13) | 86.5% (32) |
|  | **AB7** | **AS** | 49% (200) | 12.3% (69) | 1.2% (4) | 20.1% (82) | 17.4% (71) | 37.5% (153) |
|  |  | **SS** | 13.8% (4) | - (0) | - (0) | 48.3% (14) | 37.9% (11) | 86.2% (35) |
|  | **M16** | **AS** | 48.8% (185) | 21.1% (80) | 0.5% (2) | 16.4% (60) | 13.2% (50) | 29.6% (110) |
|  |  | **SS** | 15.6% (5) | 3.1% (1) | 3.1% (1) | 53.2% (17) | 25% (8) | 78.2% (25) |
|  | **Total** | **AS** | 45.3% (540) | 16.8% (201) | 0.7% (7) | 17.0% (202) | 20.2% (240) | 37.2% (442) |
|  |  | **SS** | 13.3% (13) | 1.0% (1) | 2.0% (2) | 51.0% (50) | 32.7% (32) | 83.7% (82) |
| **II-ni** | **AB2** | **AS** | 36.9% (155) | 20.2% (85) | 0.2% (1) | 18.7% (78) | 24% (101) | 42.7% (179) |
|  |  | **SS** | 3.8% (1) | - (0) | - (0) | 42.4% (11) | 53.8% (14) | 96.2% (35) |
|  | **AB7** | **AS** | 47.8% (220) | 17.2% (79) | 0.2% (1) | 18.7% (86) | 16.1% (74) | 34.8% (160) |
|  |  | **SS** | 15.4% (4) | - (0) | 3.8% (1) | 38.5% (10) | 42.3% (11) | 80.8% (21) |
|  | **M16** | **AS** | 48.8% (202) | 21.1% (57) | 0.5% (1) | 16.4% (54) | 13.2% (53) | 29.6% (107) |
|  |  | **SS** | 2.3% (1) | 2.3% (1) | 2.3% (1) | 81.5% (35) | 11.6% (5) | 93.1% (40) |
|  | **Total** | **AS** | 46.3% (577) | 17.7% (221) | 0.2% (3) | 17.4% (217) | 18.4% (229) | 35.8% (446) |
|  |  | **SS** | 6.3% (6) | 1.1% (1) | 2.1% (2) | 58.9% (56) | 31.6% (30) | 90.5% (86) |
| **III** | **AB2** | **AS** | 37.5% (135) | 21.4% (77) | - (0) | 21.4% (77) | 19.7% (71) | 41.1% (148) |
|  |  | **SS** | 5% (1) | - (0) | - (0) | 55% (11) | 40% (8) | 95% (19) |
|  | **AB7** | **AS** | 39.5% (153) | 18.1% (70) | 1% (3) | 19.2% (74) | 22.2% (86) | 41.4% (160) |
|  |  | **SS** | 10% (2) | 10% (2) | - (0) | 20% (4) | 60% (12) | 80% (16) |
|  | **M16** | **AS** | 45.5% (179) | 13.5% (53) | 0.8% (3) | 27.2% (107) | 13% (51) | 40.2% (158) |
|  |  | **SS** | 9.4% (3) | 3.1% (1) | - (0) | 68.7% (22) | 18.8% (6) | 87.5% (28) |
|  | **Total** | **AS** | 40.8% (467) | 17.5% (200) | 0.5% (6) | 22.2% (255) | 19% (218) | 41.2% (473) |
|  |  | **SS** | 8.3% (6) | 4.2% (3) | - (0) | 51.4% (37) | 36.1% (26) | 87.5% (63) |
| **Va/b** | **AB2** | **AS** | 37.5% (140) | 16.9% (63) | 1.9% (7) | 23.9% (89) | 19.8% (74) | 43.7% (163) |
|  |  | **SS** | 7.1% (1) | - (0) | 7.1% (1) | 64.4% (9) | 21.4% (3) | 85.8% (12) |
|  | **AB7** | **AS** | 46% (180) | 15.4% (60) | 0.5% (2) | 21.7% (85) | 16.4% (64) | 38.1% (149) |
|  |  | **SS** | - (0) | - (0) | 6.3% (1) | 43.7% (4) | 50.0% (12) | 93.7% (16) |
|  | **M16** | **AS** | 24.3% (67) | 22.1% (61) | 0.7% (2) | 21% (58) | 31.9% (88) | 52.9% (146) |
|  |  | **SS** | - (0) | - (0) | - (0) | 33.3% (3) | 66.7% (6) | 100% (9) |
|  | **Total** | **AS** | 37.2% (387) | 17.7% (184) | 1.1% (11) | 22.3% (232) | 21.7% (226) | 44% (458) |
|  |  | **SS** | 2.6% (1) | - (0) | 5.1% (2) | 48.7% (19) | 43.6% (17) | 92.3% (36) |
| **Vc** | **AB2** | **AS** | 31.5% (142) | 33.8% (153) | 0.7% (3) | 12.9% (58) | 21.1% (95) | 34% (153) |
|  |  | **SS** | 9.1% (2) | 9.1% (2) | - (0) | 36.4% (8) | 45.4% (10) | 81.8% (18) |
|  | **AB7** | **AS** | 40.8% (154) | 27.6% (104) | 0.8% (3) | 20.7% (78) | 10.1% (38) | 30.8% (116) |
|  |  | **SS** | 16.7% (5) | 3.3% (1) | - (0) | 46.7% (14) | 33.3% (10) | 80% (24) |
|  | **M16** | **AS** | 35.6% (124) | 24.1% (84) | 0.9% (3) | 13.5% (47) | 25.9% (90) | 39.4% (137) |
|  |  | **SS** | 26.7% (8) | - (0) | 3.3 (1) | 36.7% (11) | 33.3% (10) | 70% (21) |
|  | **Total** | **AS** | 35.7% (420) | 29% (341) | 0.8% (9) | 15.5% (183) | 19% (223) | 34.5% (406) |
|  |  | **SS** | 18.2% (15) | 3.6% (3) | 1.2% (1) | 40.3% (33) | 36.7% (30) | 77% (63) |
| **VI** | **AB2** | **AS** | 46.9% (167) | 31.2% (111) | 0.6% (2) | 11.8% (42) | 9.5% (34) | 21.3% (76) |
|  |  | **SS** | - (0) | - (0) | - (0) | 45.5% (5) | 54.5% (10) | 100% (15) |
|  | **AB7** | **AS** | 47.5% (164) | 29.0% (100) | - (0) | 9.3% (32) | 14.2% (49) | 23.5% (81) |
|  |  | **SS** | 13.3% (2) | 6.7% (1) | - (0) | 66.7% (10) | 13.3% (2) | 80% (12) |
|  | **M16** | **AS** | 50.1% (184) | 33.8% (124) | 0.3% (1) | 8.7% (32) | 7.1% (26) | 15.8% (58) |
|  |  | **SS** | - (0) | 14.2% (3) | 4.8% (1) | 42.9% (9) | 38.1% (8) | 81% (17) |
|  | **Total** | **AS** | 48.2% (515) | 31.4% (335) | 0.3% (3) | 9.9% (106) | 10.2% (109) | 20.1% (215) |
|  |  | **SS** | 4.3% (2) | 8.5% (4) | 2.1% (1) | 51.1% (24) | 34% (16) | 85.1% (40) |
| **I-VI** | **AB2** | **AS** | 38.4% (1054) | 22.2% (609) | 0.5% (15) | 17.1% (471) | 21.8% (598) | 38.9% (1069) |
|  |  | **SS** | 5.7% (9) | 1.3% (2) | 1.8% (3) | 42.8% (68) | 48.4% (77) | 91.2% (145) |
|  | **AB7** | **AS** | 44.3% (1227) | 19.2% (532) | 0.7% (18) | 18.2% (503) | 17.6% (487) | 35.8% (990) |
|  |  | **SS** | 10.8% (17) | 5.7% (9) | 1.9% (3) | 42% (66) | 39.6% (62) | 81.6% (128) |
|  | **M16** | **AS** | 43.5% (1099) | 21.4% (541) | 0.5% (12) | 17.4% (439) | 17.2% (433) | 34.6% (872) |
|  |  | **SS** | 9.3% (18) | 3.6% (7) | 2.6% (5) | 56% (108) | 28.5% (55) | 84.5% (163) |
|  | **Total** | **AS** | 42.1% (3380) | 20.9% (1682) | 0.5% (45) | 17.6% (1413) | 18.9% (1518) | 36.5% (2931) |
|  |  | **SS** | 8.6% (44) | 3.5% (18) | 2.2% (11) | 47.5% (242) | 38.2% (194) | 85.7% (436) |

Supplementary File 1k. Proportion of the post-synaptic targets of synaptic junctions in MEC layers, for individual cases. Total synapses on spine heads are calculated as the sum of “Complete Spine Heads” and “Incomplete Spine Heads” columns. Data in parentheses refer to the absolute numbers of synapses.

| **Layer** | **Type of Synapse** | **Spine Head** | **Spine Neck** | **Spiny Shaft** | **Aspiny Shaft** | **Shafts**  **(Spiny + Aspiny)** |
| --- | --- | --- | --- | --- | --- | --- |
| **I** | **AS** | 119,082 ± 6,100 (110,747 ± 5,673) | 68,168 ± 18,563 (62,396 ± 17,263) | 101,986 ± 5,371 (94,847 ± 4,995) | 98,581 ± 4,375 (91,680 ± 4,069) | 99,960 ± 3,545 (92,962 ± 3,297) |
|  | **SS** | 161,971 (150,633) | 42,755 ± 3,477 (39,762 ± 3,234) | 48,287 ± 6,885 (44,907 ± 6,403) | 46,804 ± 3,553 (43,528 ± 3,305) | 48,094 ± 2,042 (44,727 ± 1,899) |
| **II-is** | **AS** | 125,834 ± 6,599 (117,025 ± 6,137) | 78,709 ± 20,174 (73,200 ± 18,761) | 117,234 ± 6,063 (109,028 ± 5,639) | 110,359 ± 7,633 (102,634 ± 7,098) | 115,956 ± 5,572 (107,839 ± 5,182) |
|  | **SS** | 68,081 ± 11,271 (63,316 ± 10,482) | 70,880 ± 4,112 (65,919 ± 3,824) | 72,899 ± 7,931 (67,796 ± 7,376) | 67,660 ± 6,457 (62,924 ± 6,005) | 69,017 ± 5,549 (64,186 ± 5,160) |
| **II-ni** | **AS** | 124,614 ± 8,464 (115,891 ± 7,872) | 69,252 ± 3,827 (64,404 ± 3,559) | 138,955 ± 6,692 (129,228 ± 6,224) | 108,619 ± 8,056 (101,016 ± 7,493) | 124,575 ± 6,706 (115,854 ± 6,237) |
|  | **SS** | 58,746 ± 25,087 (54,633 ± 23,331) | 43,048 ± 5,728 (40,035 ± 5,327) | 74,894 ± 9,012 (69,651 ± 8,381) | 72,447 ± 10,324 (67,376 ± 9,601) | 77,563 ± 8,752 (72,134 ± 8,140) |
| **III** | **AS** | 143,154 ± 7,462 (133,133 ± 6,940) | 69,862 ± 18,204 (64,972 ± 16,929) | 139,844 ± 8,879 (130,055 ± 8,258) | 127,158 ± 8,187 (118,257 ± 7,641) | 131,819 ± 7,258 (122,591 ± 6,750) |
|  | **SS** | 112,999 ± 46,011 (105,089 ± 42,791) | - | 82,262 ± 11,026 (76,503 ± 10,255) | 66,073 ± 9,339 (61,448 ± 8,685) | 72,577 ± 8,570 (67,496 ± 7,970) |
| **Va/b** | **AS** | 148,730 ± 7,348 (138,319 ± 7,348) | 67,245 ± 16,663 (62,537 ± 15,497) | 151,189 ± 10,398 (140,606 ± 9,670) | 137,209 ± 5,457 (127,604 ± 5,075) | 143,122 ± 6,939 (133,104 ± 6,453) |
|  | **SS** | 71,678 (66,660) | 32,816 ± 20,518 (30,519 ± 19,082) | 59,604 ± 5,089 (55,432 ± 4,733) | 83,131 ± 12,141 (77,312 ± 11,291) | 67,851 ± 6,157 (63,101 ± 5,726) |
| **Vc** | **AS** | 128,645 ± 6,101 (119,346 ± 5,673) | 60,443 ± 8,584 (56,212 ± 7,983) | 135,639 ± 5,381 (126,144 ± 5,004) | 131,950 ± 7,869 (122,714 ± 7,319) | 127,584 ± 5,012 (118,653 ± 4,661) |
|  | **SS** | 79,588 ± 10,548 (74,017 ± 9,809) | 115,738 (107,636) | 96,546 ± 10,923 (89,788 ± 10,158) | 86,027 ± 4,670 (80,005 ± 4,343) | 85,941 ± 6,115 (79,926 ± 5,687) |
| **VI** | **AS** | 105,948 ± 8,586 (98,532 ± 7,985) | 48,950 ± 6,519 (45,524 ± 6,063) | 99,751 ± 12,528 (92,769 ± 11,651) | 105,833 ± 10,656 (98,424 ± 9,910) | 103,833 ± 9,881 (96,564 ± 9,189) |
|  | **SS** | 33,254 ± 18,289 (30,918 ± 17,009) | 52,193 (48,540) | 96,837 ± 11,956 (90,058 ± 11,119) | 51,591 ± 11,093 (47,980 ± 10,317) | 70,343 ± 10,522 (65,419 ± 9,786) |
| **I-VI** | **AS** | 127,956 ± 3,120 (118,999 ± 2,902) | 66,435 ± 5,616 (61,784 ± 5,223) | 126,371 ± 3,808 (117,525 ± 3,542) | 117,101 ± 3,266 (108,904 ± 3,038) | 120,978 ± 3,006 (112,510 ± 2,796) |
|  | **SS** | 76,801 ± 9,326 (71,425 ± 8,673) | 53,608 ± 7,928 (49,855 ± 7,373) | 76,403 ± 3,959 (71,055 ± 3,681) | 68,026 ± 3,560 (63,264 ± 3,311) | 70,198 ± 2,948 (65,284 ± 2,742) |

Supplementary File 1l. SAS area of asymmetric (AS) and symmetric (SS) synapses regarding the post-synaptic target, in each MEC layer. Data in parentheses are not corrected for shrinkage. Values (in nm^2^) are expressed as mean ± standard error.

| **Layer** | **Case** | **Type of Synapse** | **Spine Head** | **Spine Neck** | **Spiny Shaft** | **Aspiny Shaft** | **Shafts**  **(Spiny + Aspiny)** |
| --- | --- | --- | --- | --- | --- | --- | --- |
| **I** | **AB2** | **AS** | 120,467 ± 9,430 (112,034 ± 8,770) | 92,839 (86,340) | 118,246 ± 6,221 (109,969 ± 5,785) | 97,256 ± 8,078 (90,448 ± 3,157) | 105,655 ± 2,639 (98,259 ± 2,454) |
|  |  | **SS** | - | 43,604 (40,552) | 43,049 ± 16,837 (40,035 ± 15,658) | 45,416 ± 1,848 (42,237 ± 1,719) | 47,413 ± 2,243 (44,094 ± 2,086) |
|  | **AB7** | **AS** | 115,304 ± 7,209 (107,232 ± 6,705) | 55,833 ± 24,026 (51,924 ± 2,344) | 89,724 ± 5,185 (83,444 ± 4,822) | 100,994 ± 9,662 (93,924 ± 8,986) | 96,381 ± 5,605 (89,634 ± 5,213) |
|  |  | **SS** | - | 36,352 (33,808) | 52,413 ± 8,390 (48,744 ± 7,803) | 50,674 ± 6,169 (47,127 ± 5,737) | 49,327 ± 5,114 (45,874 ± 4,756) |
|  | **M16** | **AS** | 121,477 ± 17,164 (108,683 ± 15,962) | - | 97,989 ± 8,078 (91,130 ± 7,513) | 97,493 ± 10,973 (90,669 ± 10,205) | 97, 843 ± 9,358 (90,994 ± 8,703) |
|  |  | **SS** | 161,971 (150,633) | 48,307 (44,926) | 52,017 ± 1,577 (48,376 ± 1,467) | 44,322 ± 9,926 (41,219 ± 9,231) | 47,541 ± 4,210 (44,213 ± 3,916) |
| **II-is** | **AB2** | **AS** | 150,618 ± 4,582 (140,074 ± 4,261) | 158,471 (147,378) | 132,635 ± 6,564 (123,351 ± 6,105) | 138,754 ± 2,885 (129,041 ± 2,683) | 136,821 ± 4,259 (127,243 ± 3,961) |
|  |  | **SS** | 88,885 ± 26,925 (82,663 ± 25,040) | 66,768 (62,095) | 85,382 ± 16,411 (79,405 ± 15,262) | 70,965 ± 4,789 (65,997 ± 4,454) | 76,556 ± 6,632 (71,197 ± 6,167) |
|  | **AB7** | **AS** | 109,826 ± 4,449 (102,138 ± 4,138) | 54,651 ± 7,680 (50,826 ± 7,142) | 110,825 ± 8,221 (103,067 ± 7,645) | 95,052 ± 3,873 (88,398 ± 3,602) | 103,746 ± 3,556 (96,484 ± 3,307) |
|  |  | **SS** | 55,540 ± 4,129 (51,652 ± 3,840) | - | 57,479 ± 5,862 (53,456 ± 5,451) | 49,167 ± 3,658 (45,725 ± 3,402) | 54,719 ± 3,688 (50,888 ± 3,430) |
|  | **M16** | **AS** | 117,058 ± 2,832 (108,864 ± 2,634) | 62,887 ± 827 (58,485 ± 769) | 108,243 ± 2,285 (100,666 ± 11,425) | 97,272 ± 8,356 (90,463 ± 7,771) | 107,303 ± 3,481 (99,791 ± 3,237) |
|  |  | **SS** | 55,689 ± 17,096 (51,791 ± 15,900) | 74,992 (69,742) | 75,836 ± 15,812 (70,528 ± 14,705) | 90,443 ± 3,183 (84,112 ± 2,960) | 75,776 ± 12,585 (70,472 ± 11,704) |
| **II-ni** | **AB2** | **AS** | 134,313 ± 18,124 (124,912 ± 16,855) | 76,356 (71,011) | 142,178 ± 3,698 (132,226 ± 12,739) | 113,555 ± 3,135 (105,606 ± 2,915) | 124,586 ± 2,642 (115,865 ± 2,457) |
|  |  | **SS** | 158,933 (147,808) | - | 78,060 ± 22,575 (72,596 ± 20,995) | 65,493 ± 5,453 (60,909 ± 5,071) | 71,043 ± 6,070 (66,070 ± 5,645) |
|  | **AB7** | **AS** | 107,195 ± 6,397 (99,691 ± 5,950) | 68,166 (63,395) | 127,407 ± 14,624 (118,489 ± 13,600) | 80,975 ± 8,544 (75,306 ± 7,946) | 108,497 ± 15,681 (100,902 ±14,583) |
|  |  | **SS** | 34,529 ± 2,313 (32,112 ± 2,151) | 37,320 (34,708) | 55,050 ± 1,687 (51,197 ± 1,569) | 74,271 ± 30,748 (69,072 ± 28,596) | 78,389 ± 28,654 (72,902 ± 26,648) |
|  | **M16** | **AS** | 132, 334 ± 16,181 (123,071 ± 15,048) | 63,233 (58,807) | 147,279 ± 5,426 (136,970 ± 5,046) | 131,328 ± 6,627 (122,135 ± 6,163) | 140,641 ± 5,337 (130,796 ± 4,963) |
|  |  | **SS** | 31,209 (29,024) | 48,777 (45,363) | 84,956 ± 7,781 (79,009 ± 7,237) | 77,577 ± 16,274 (72,147 ± 15,135) | 83,259 ± 4,851 (77,431 ± 4,511) |
| **III** | **AB2** | **AS** | 147,924 ± 9,132 (140,074 ± 4,261) | - | 150,023 ± 15,852 (139,521 ± 14,742) | 145,951 ± 6,739 (135,734 ± 6,267) | 148,328 ± 11,445 (137,945 ± 10,644) |
|  |  | **SS** | 249,328 (231,875) | - | 101,092 ± 10,065 (94,015 ± 9,360) | 82,422 ± 16,741 (76,653 ± 15,569) | 87,154 ± 1,548 (81,053 ± 1,440) |
|  | **AB7** | **AS** | 143,059 ± 17,823 (130,782 ± 16,576) | 60,085 ± 26,927 (55,879 ± 25,042) | 142,772 ± 22,265 (132,778 ± 20,706) | 98,564 ± 10,477 (91,665 ± 9,744) | 117,208 ± 14,761 (109,003 ± 13,728) |
|  |  | **SS** | 54,269 (50,470) | - | 56,503 ± 775 (52,547 ± 721) | 51,868 ± 15,681 (48,238 ± 14,583) | 52,796 ± 8,592 (49,100 ± 7,991) |
|  | **M16** | **AS** | 138,478 ± 15,646 (122,066 ± 14,551) | 80,726 ± 23,662 (75,075 ± 22,006) | 126,737 ± 7,554 (117,866 ± 7,025) | 136,958 ± 3,963 (127,371 ± 3,686) | 129,920 ± 6,195 (120,825 ± 5,761) |
|  |  | **SS** | 74,200 ± 13,394 (69,006 ± 12,457) | - | 80,604 ± 25,168 (74,962 ± 23,406) | 55,753 ± 5,973 (51,850 ± 5,555) | 77,780 ± 22,130 (72,336 ± 20,581) |
| **Va/b** | **AB2** | **AS** | 166,753 ± 8,190 (155,081 ± 7,617) | 80,201 ± 11,699 (74,587 ± 10,880) | 165,654 ± 4,971 (154,058 ± 4,623) | 150,719 ± 7,462 (140,169 ± 6,939) | 158,490 ± 5,898 (147,395 ± 5,486) |
|  |  | **SS** | 71,678 (66,660) | 12,298 (11,437) | 63,876 ± 6,495 (59,405 ± 6,041) | 76,063 ± 25,752 (70,739 ± 23,950) | 67,725 ± 5,624 (62,984 ± 5,230) |
|  | **AB7** | **AS** | 127,985 ± 10,154 (119,026 ± 9,443) | 101,976 ± 32,066 (94,837 ± 29,821) | 123,017 ± 13,268 (114,406 ± 12,339) | 122,163 ± 5,284 (113,612 ± 4,915) | 123,224 ± 9,182 (114,598 ± 8,539) |
|  |  | **SS** | - | 53,334 (49,601) | 59,023 ± 13,066 (54,891 ± 12,151) | 98,873 ± 22,517 (91,952 ± 20,940) | 77,956 ± 15,833 (72,499 ± 14,724) |
|  | **M16** | **AS** | 151,451 ± 9,819 (140,850 ± 9,131) | 13,079 ± 504 (12,164 ± 469) | 164,896 ± 22,389 (153,353 ± 20,822) | 138,745 ± 8,249 (129,032 ± 7,672) | 147,653 ± 11,503 (137,317 ± 10,698) |
|  |  | **SS** | - | - | 55,914 ± 9,046 (52,000 ± 8,412) | 70,119 ± 893 (65,210 ± 831) | 57,871 ± 8,473 (53,820 ± 7,880) |
| **Vc** | **AB2** | **AS** | 139,246 ± 8,631 (129,499 ± 8,027) | 56,969 ± 12,572 (52,981 ± 11,692) | 136,775 ± 10,553 (127,200 ± 9,814) | 128,880 ± 4,273 (119,858 ± 3,974) | 131,651 ± 6,311 (122,436 ± 5,869) |
|  |  | **SS** | 48,887 ± 2,496 (45,465 ± 2,321) | - | 90,867 ± 17,900 (84,506 ± 16,647) | 80,884 ± 11,685 (75,222 ± 10,867) | 88,640 ± 8,926 (82,435 ± 8,302) |
|  | **AB7** | **AS** | 117,786 ± 9,871 (109,541 ± 9,180) | 52,166 ± 26,276 (48,514 ± 24,437) | 129,878 ± 6,230 (120,786 ± 5,794) | 143,514 ± 23,352 (133,468 ± 21,718) | 132,248 ± 13,548 (122,991 ± 12,600) |
|  |  | **SS** | 84,649 ± 18,704 (78,723 ± 17,395) | - | 102,861 ± 22,263 (95,660 ± 20,705) | 88,327 ± 9,312 (82,144 ± 8,660) | 91,241 ± 15,165 (84,854 ± 14,103) |
|  | **M16** | **AS** | 127,954 ± 12,626 (118,997 ± 11,742) | 73,930 ± 10,687 (68,755 ± 9,939) | 140,264 ± 13,012 (130,446 ± 12,101) | 123,457 ± 8,479 (114,815 ± 7,886) | 126,953 ± 7,026 (118,066 ± 6,534) |
|  |  | **SS** | 94,996 ± 15,517 (88,346 ± 14,430) | 115,738 (107636) | 95,910 ± 24,071 (89,197 ± 22,386) | 88,869 ± 4,303 (82,648 ± 4,002) | 94,847 ± 8,257 (88,208 ± 7,679) |
| **VI** | **AB2** | **AS** | 123,846 ± 17,263 (115,176 ± 16,055) | 55,467 ± 295 (51,585 ± 274) | 98,618 ± 19,859 (91,715 ± 18,469) | 134,501 ± 12,197 (125,086 ± 11,343) | 117,723 ± 12,160 (109,482 ± 11,309) |
|  |  | **SS** | - | - | 118,882 ± 22,081 (110,560 ± 20,535) | 36,961 ± 22,025 (34,374 ± 20,484) | 81,725 ± 22,427 (76,004 ± 20,857) |
|  | **AB7** | **AS** | 79,798 ± 3,452 (74,212 ± 3,211) | - | 65,899 ± 11,109 (61,286 ± 10,332) | 69,969 ± 6,480 (65,072 ± 6,026) | 69,715 ± 5,101 (64,835 ± 4,744) |
|  |  | **SS** | 33,245 ± 18,289 (30,918 ± 17,009) | - | 55,862 ± 1,095 (51,952 ± 1,018) | 29,850 ± 10,639 (27,760 ± 9,894) | 42,965 ± 11,940 (39,958 ± 11,104) |
|  | **M16** | **AS** | 114,201 ± 6,212 (106,207 ± 5,777) | 35,917 (33,402) | 134,737 ± 13,417 (125,306 ± 12,478) | 113,028 ± 12,160 (105,116 ± 8,921) | 124,061 ± 10,709 (115,377 ± 9,959) |
|  |  | **SS** | - | 52,193 (48,540) | 102,108 ± 5,441 (94,960 ± 5,061) | 75,839 ± 11,794 (70,531 ± 10,968) | 86,337 ± 10,752 (80,294 ± 10,000) |

**Supplementary File 1m. SAS area of asymmetric (AS) and symmetric (SS) synapses according to the post-synaptic target in each MEC layer, per individual case.** Data in parentheses are not corrected for shrinkage. Values (in nm^2^) are expressed as mean ± standard error.

| **Layer** | **% CV Synaptic density** | **% CV Proportion of AS** | **% CV AS SAS area** | **% CV Proportion of macular AS** | **% CV Proportion of AS on spines** |
| --- | --- | --- | --- | --- | --- |
| **I** | 12.2 | 1.4 | 10.2 | 6.8 | 5.7 |
| **II-is** | 11.0 | 2.1 | 15.8 | 3.5 | 9.1 |
| **II-ni** | 12.9 | 3.6 | 14.2 | 5.2 | 15.6 |
| **III** | 18.7 | 2.7 | 13.2 | 11.2 | 11.0 |
| **Va/b** | 13.9 | 1.3 | 12.3 | 7.8 | 17.8 |
| **Vc** | 20.4 | 2.4 | 12 | 3.6 | 12.1 |
| **VI** | 14.8 | 2.2 | 19.4 | 5.3 | 4.4 |

**Supplementary File 1n. Coefficient of Variation of the analyzed synaptic parameters in each MEC layer between the stack of images.** AS: asymmetric synapses; CV: coefficient of variation; SAS: synaptic apposition surface.

| **Region / Layer** | **Animal** | **Reference** | **Microscopy** | **No. Case/Sex/Age** | **AS**  **Spine Heads (%)** | **AS Spine Necks (%)** | **AS**  **Spiny Shafts (%)** | **AS Aspiny Shafts (%)** | **SS Spine Heads (%)** | **SS Spine Necks (%)** | **SS Spiny Shafts (%)** | **SS Aspiny Shafts (%)** |
| --- | --- | --- | --- | --- | --- | --- | --- | --- | --- | --- | --- | --- |
| **CEM**  **I-VI** | Human | Present Study | FIB/SEM | 2M 1F  (40-66) | 59.3 | 0.5 | 16.5 | 17.8 | 0.7 | 0.1 | 2.8 | 2.3 |
| **CE**  **II** | Human | Domínguez-Álvaro et al., 2021 | FIB/SEM | 4M  (40-66) | 53.8 | 0.5 | 19.3 | 18.2 | 1.2 | 0.1 | 5.1 | 1.9 |
| **CE**  **III** | Human | Domínguez-Álvaro et al., 2021 | FIB/SEM | 4M  (40-66) | 51 | 0.7 | 24.8 | 16.2 | 1.2 | 0.2 | 4.2 | 1.6 |
| **CTE**  **II** | Human | Domínguez-Álvaro et al., 2019 | FIB/SEM | 4M 1F  (36-66) | 54.7 | 0.5 | 19 | 18.4 | 0.5 | 0.1 | 4 | 2.8 |
| **BA21**  **III** | Human | Cano-Astorga et al., 2023 | FIB/SEM | 2M 1F  (53-66) | 69.4 | 1.1 | 11.8 | 10.8 | 1.2 | 0.3 | 3.4 | 2.4 |
| **BA24**  **III** | Human | Cano-Astorga et al., 2023 | FIB/SEM | 2M 1F  (53-66) | 68.5 | 1 | 13.2 | 10.8 | 1.3 | 0.6 | 2.2 | 2.5 |
| **vBA38**  **III** | Human | Cano-Astorga et al., 2023 | FIB/SEM | 2M 1F  (53-66) | 73.4 | 0.6 | 10.6 | 10.2 | 1.2 | 0.3 | 2.6 | 1.1 |
| **dBA38**  **III** | Human | Cano-Astorga et al., 2023 | FIB/SEM | 2M 1F  (53-66) | 71.3 | 0.8 | 11.9 | 10.1 | 1.1 | 0.3 | 2.6 | 1.9 |
| **CA1**  **(All Layers)** | Human | Montero-Crespo et al., 2020 | FIB/SEM | 3M 2F  (36-65) | 77.8 | 0.2 | 5.6 | 5.8 | 0.9 | 0.2 | 6.2 | 5.8 |
| **Temporal Cortex**  **V** | Human | Yakuobi et al., 2019 | TEM | 3M 4F  (20-50) | 85* | - | 15*(*) | - | - | - | - | - |
| **Temporal Cortex**  **VI** | Human | Schmuhl-Giesen et al., 2022 | TEM | 2M 2F  (25-63) | 80* | - | 20*(*) | - | - | - | - | - |
| **Somatosensory Cortex**  **I-VI** | Mouse | Turégano-López, 2022 | FIB/SEM | 3M  (8 weeks old) | 74.1 | 2 | 10.3 | 2.3 | 3.55 | - | 7.4 | 0.5 |
| **Somatosensory Cortex**  **I-VI** | Etruscan Shrew | Alonso-Nanclares et al., 2023 | FIB/SEM | 3M  (8-20 months old) | 77.9*** | - | 13.9** | - | 0.8*** | - | 7.1** | - |
| **Somatosensory Cortex**  **I-VI** | Juvenile Rat | Santuy et al., 2018 | FIB/SEM | 3M  (14 days old) | 73.9 | 1.6 | 14.7*** | - | 2 | 0.7 | 7*** | - |

**Supplementary File 1o. Data on postsynaptic targets in different species, regions and cortical layers.** Abbreviations: EC: Entorhinal Cortex; F: Female; FIB/SEM: Focused Ion Beam-Scanning Electron Microscopy; M: Male; MEC: Medial Entorhinal Cortex; TCE: Transentorhinal Cortex; TEM: Transmission Electron Microscopy. Age refers to years, except otherwise indicated. *No classification of the type of synapses (AS:SS) were performed. **Only axospinous synapses (established on dendritic spine heads and necks) and axodendritic synapses (formed on dendritic shafts) are indicated. ***No distinction between spiny and aspiny shafts were made. See references below

References for Supplementary File 1o

Alonso-Nanclares L, Rodríguez JR, Merchan-Perez A, González-Soriano J, Plaza-Alonso S, Cano-Astorga N, Naumann RK, Brecht M, DeFelipe J. 2023. Cortical synapses of the world’s smallest mammal: An FIB/SEM study in the Etruscan shrew. The Journal of Comparative Neurology 531:390–414. Doi: https://doi.org/10.1002/cne.25432 10.1002/cne.25432

Cano-Astorga N, Plaza-Alonso S, DeFelipe J, Alonso-Nanclares L. 2023. 3D synaptic organization of layer III of the human anterior cingulate and temporopolar cortex. Cerebral Cortex 33:9691–9708. Doi: 10.1093/cercor/bhad232

Domínguez-Álvaro M, Montero-Crespo M, Blazquez-Llorca L, DeFelipe J, Alonso-Nanclares L. 2019. 3D Electron Microscopy Study of Synaptic Organization of the Normal Human Transentorhinal Cortex and Its Possible Alterations in Alzheimer’s Disease. eNeuro 6:ENEURO.0140-19.2019. Doi: 10.1523/ENEURO.0140-19.2019

Domínguez-Álvaro M, Montero-Crespo M, Blazquez-Llorca L, Plaza-Alonso S, Cano-Astorga N, DeFelipe J, Alonso-Nanclares L. 2021. 3D analysis of the synaptic organization in the entorhinal cortexAnalysis of the Synaptic Organization in the Entorhinal Cortex in Alzheimer’s disease. eNeuro 8:ENEURO.0504-20.2021. Doi: 10.1523/ENEURO.0504-20.2021

Montero-Crespo M, Dominguez-Alvaro M, Rondon-Carrillo P, Alonso-Nanclares L, DeFelipe J, Blazquez-Llorca L. 2020. Three-dimensional synaptic organization of the human hippocampal CA1 field. eLife 9:e57013. Doi: 10.7554/eLife.57013

Santuy A, Rodriguez JR, DeFelipe J, Merchan-Perez A. 2018. Volume electron microscopy of the distribution of synapses in the neuropil of the juvenile rat somatosensory cortex. Brain Structure & Function 223:77–90. Doi: https://doi.org/10.1007/s00429-017-1470-7 10.1007/s00429-017-1470-7

Schmuhl-Giesen S, Rollenhagen A, Walkenfort B, Yakoubi R, Sätzler K, Miller D, von Lehe M, Hasenberg M, Lübke JHR. 2022. Sublamina-Specific Dynamics and Ultrastructural Heterogeneity of Layer 6 Excitatory Synaptic Boutons in the Adult Human Temporal Lobe Neocortex. Cerebral Cortex 32:1840–1865. Doi: <https://doi.org/10.1093/cercor/bhab31510.1093/cercor/bhab315>

Turégano-López M. 2022. Ultraestructura y conectividad de la corteza cerebral (Doctoral dissertation, Autonomous University of Madrid). Repositorio de Datos de Investigación UAM. <http://hdl.handle.net/10486/705257>

Yakoubi R, Rollenhagen A, von Lehe M, Shao Y, Sätzler K, Lübke JHR. 2019. Quantitative Three-Dimensional Reconstructions of Excitatory Synaptic Boutons in Layer 5 of the Adult Human Temporal Lobe Neocortex: A Fine-Scale Electron Microscopic Analysis. Cerebral Cortex 29:2797–2814. Doi: https://doi.org/10.1093/cercor/bhy14610.1093/cercor/bhy146

| **Case** | **Sex** | **Age (years)** | **Cause of death** | **Post-mortem delay (h)** | **Neurological diagnosis** |
| --- | --- | --- | --- | --- | --- |
| AB2 | Female | 53 | Pulmonary Shock | 4 | No neurological alterations |
| AB7 | Male | 66 | Metastatic Bladder Carcinoma | 2.4 | No neurological alterations |
| M16 | Male | 40 | Car Accident | 3 | No neurological alterations |

Supplementary File 1p. Clinical and neuropsychological information from the cases analyzed.
